# Protective Effect of Surfactant Protein D in Pulmonary Vaccinia Virus Infection: Implication of A27 Viral Protein

**DOI:** 10.3390/v5030928

**Published:** 2013-03-21

**Authors:** Perino Julien, Nicole M. Thielens, Erika Crouch, Danièle Spehner, Jean-Marc Crance, Anne-Laure Favier

**Affiliations:** 1 Laboratoire de Virologie, Institut de Recherche Biomédicale des Armées- Antenne du Centre de Recherches du Service de Santé des Armées, 38702 La Tronche cedex, France; E-Mails: julien0012000@yahoo.fr (J.P.); jeanmarccrance@orange.fr (J-M.C.); favier03al@yahoo.fr (A-L.F.); 2 Institut de Biologie Structurale, CNRS, CEA, Université Joseph Fourier, Grenoble, France; E-Mail: nicole.thielens@ibs.fr (N-M.T.); 3 Dept of Pathology and Immunology, Washington University School of Medicine, Saint Louis, MO, USA; E-Mail: crouch@wustl.edu (E.C.); 4 IGBMC; CNRS, UMR 7104; Inserm U 596; Illkirch, F-67400 France; Université Louis Pasteur, Strasbourg, F-67000 France; E-Mail: daniele.spehner@igbmc.u-strasbg.fr (D.S.)

**Keywords:** orthopoxvirus, surfactant protein D, vaccinia virus, A27 protein, pulmonary infection, innate immunity, surfactant protein A, KO mice, Ca^2+^, sugars

## Abstract

Vaccinia virus (VACV) was used as a surrogate of variola virus (VARV) (genus *Orthopoxvirus*), the causative agent of smallpox, to study Orthopoxvirus infection. VARV is principally transmitted between humans by aerosol droplets. Once inhaled, VARV first infects the respiratory tract where it could encounter surfactant components, such as soluble pattern recognition receptors. Surfactant protein D (SP-D), constitutively present in the lining fluids of the respiratory tract, plays important roles in innate host defense against virus infection. We investigated the role of SP-D in VACV infection and studied the A27 viral protein involvement in the interaction with SP-D. Interaction between SP-D and VACV caused viral inhibition in a lung cell model. Interaction of SP-D with VACV was mediated by the A27 viral protein. Binding required Ca^2+^ and interactions were blocked in the presence of excess of SP-D saccharide ligands. A27, which lacks glycosylation, directly interacted with SP-D. The interaction between SP-D and the viral particle was also observed using electron microscopy. Infection of mice lacking SP-D (SP-D^-/-^) resulted in increased mortality compared to SP-D^+/+^ mice. Altogether, our data show that SP-D participates in host defense against the vaccinia virus infection and that the interaction occurs with the viral surface protein A27.

## 1. Introduction

Vaccinia virus (VACV) is the prototype for the *Poxviridae* family and the *Orthopoxvirus* genus which includes variola virus (VARV), the etiological agent of smallpox. Since the eradication of smallpox in 1980, vaccination was discontinued [1], resulting in a large proportion of the world population susceptible to infection and heightened concerns about the threat of VARV as a bioweapon [2]. VARV could easily be aerosolized and used for bioterrorism with significant morbidity and mortality. VARV is principally transmitted between humans by aerosol droplets. Once inhaled, VARV first infects the upper- or lower-respiratory-tract mucosa and can lead to severe systemic disease, inducing death [3,4,5]. However, 70% of exposed individuals do not die of VARV infection, emphasizing the importance of the immune response and possibly the innate immune defenses against this pathogen.

**Scheme 1:**
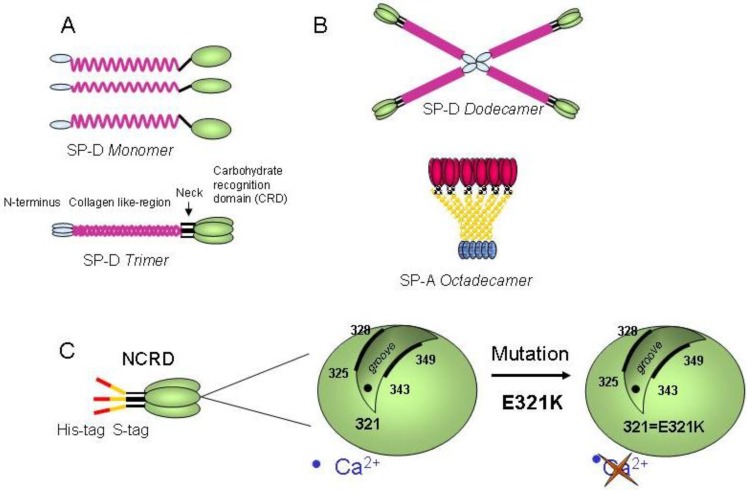
Structure of SP-D and E321K SP-D NCRD mutant. (A) SP-D monomer (illustrating CRD, neck, collagen-like and N-terminus domains) and SP-D trimer formed by three monomers. (B) SP-D dodecamer formed by four trimers linked at the N-terminus by disulfide bonds. (C) Illustration of Neck+CRD (NCRD) with a genetically engineered S protein binding tag and His binding tag. CRD cartoons (green) representing the carbohydrate binding groove flanked by amino acid residues 325–328 and 343–349, associated Ca^2+^ ion (residue 321). Scheme adapted from [50].

The alveolar spaces and airways of the lung are lined by surfactant, which contains protein and lipid components [6,7]. One lung protein of particular interest is surfactant protein D (SP-D), an important innate immune effector with known roles in antiviral host defense to airway pathogens [8]. SP-D is a member of the collectin subfamily of C-type lectins assembled from subunits comprising a triple helical collagen domain and a C-terminal globular carbohydrate recognition domain (CRD) (Scheme 1A). This trimeric subunit can multimerize into assemblies of four or more trimers. In humans, the collectin proteins also include surfactant protein A (SP-A) (Scheme 1B) and serum mannan-binding-lectin (MBL) [8,9], and show protein domain homologies to other complement recognition proteins (L-, H- and M-ficolins). As a soluble collectin secreted into the airspaces, SP-D is mostly produced by two types of pulmonary epithelial cells, alveolar type II cells and Clara cells and participates actively in host defense when assembled as multimers. SP-D immune activity [10,11] results from its pattern recognition activity towards multiple ligands present on bacteria, fungi, or viruses [12,13,14,15]. 

Binding requires the SP-D CRD and Ca^2+^, and SP-D can bind to a variety of carbohydrates in addition to the N-linked glycans of glycoproteins [13,16,17]. High affinity binding to saccharide ligands requires trimerization of the CRD, which is mediated by the contiguous neck domain [18]. Binding to certain ligands can be inhibited by saccharide ligands, even though the interactions do not appear to be mediated by the carbohydrate binding activity of SP-D [19,20]. In addition, SP-D binds also to fatty acids in a Ca^2+^-dependent manner, and binding is inhibited by glucose. Although not explained, this could reflect overlapping binding sites for carbohydrate and non-carbohydrate ligands [21]. 

This specific activity ultimately leads to opsonization, agglutination and clearance of pathogens *via* interaction with immune cells [22]. Protective roles of SP-D against various viral pathogens have been extensively studied, as for *Influenza* A virus (IAV) and respiratory syncytial virus (RSV) [23,24,25,26,27,28,29,30]. At present, there is no evidence for the involvement of the lung collectins in innate host defense against VACV.

Lung collectins are soluble pattern recognition receptors, previously demonstrated to interact with fusion proteins from different lung viral pathogens. RSV G and F glycoproteins are involved in binding of SP-D to RSV [26]. Similarly in the case of IAV, SP-D binds to the hemagglutinin (HA) [29,31,32] by interacting with the carbohydrate residues on some IAV and eventually leading to pathogen inactivation and clearance [33]. Inhibition by SP-D correlates with the presence of several glycan attachment sites on HA. Pandemic and avian strains appear to lack susceptibility to SP-D, which contributes to their virulence. IAV expressing the HA of pandemic viruses were associated with significant pathology of the lower respiratory tract and showed a low binding activity for SP-D while virus expressing HA of a seasonal strain induced only mild disease and exhibited strong *in vitro* binding to SP-D [29,34]. These studies established that the innate immune activity of SP-D is principally mediated through interaction with viral membrane glycoproteins. Vaccinia virus A27 membrane protein (also named 14-kDa fusion protein), locates on the surface of the intracellular mature virus (IMV) [35], plays an important role in virus-to-cell and cell-to-cell fusions [36,37,38]. This highly antigenic protein, involved in virulence of VACV, is conserved among *Orthopoxvirus* genus, and elicits neutralization antibodies [39]. A27 is involved in virus attachment to cell by binding glycosaminoglycans [40,41] or sulfatide [42]. Interaction with GAGs was mediated by the negative charge of the sulfate [40] that bound a stretch of positive amino acids of A27 (Lys/Arg-rich domain, residues 21-31) [41,43]. A27 was shown to have functional properties similar to those of HA of IAV, except that the transmembrane domain is replaced by a domain involved in binding to A17 protein for membrane anchoring [44,45]. The two proteins, localized on the virus surface, form trimers in the virus particle [35] and are involved in virus penetration. The NH_2_-terminal region of A27 protein is implicated in virus entry by direct fusion of the virus membrane with the cell plasma membrane [38,41] or by receptor-mediated endocytosis followed by a low pH-induced fusion of viral and endosomal membranes [46]. Accordingly, we hypothesized that SP-D might interact with A27 and participate in the pulmonary defenses against VACV. 

In addition to neutralizing virus *in vitro*, SP-D can enhance clearance *in vivo*. SP-D interacts with influenza virus [47] resulting in the increase of the virus clearance from the lung of infected animals [48,49]. Mice lacking SP-D (SP-D^-/-^) and wild type mice (SP-D^+/+^) were used as a model of infection to clarify the relevance of these various *in vitro* studies to the physiological situation.

In the current paper, we studied the effect of collectins and particularly SP-D on VACV infection *in vitro* and specifically the effect of SP-D *in vivo*. We demonstrated that SP-D was able to reduce VACV infection and showed that the A27 virus protein was a potential partner of SP-D interaction. This study represents a first approach to the involvement of SP-D in *Orthopoxvirus* infection and we suggest that SP-D may be associated with innate defense against VACV.

## 2. Results and Discussion

### 2.1. Vaccinia virus interaction with surfactant protein D

**Figure 1:**
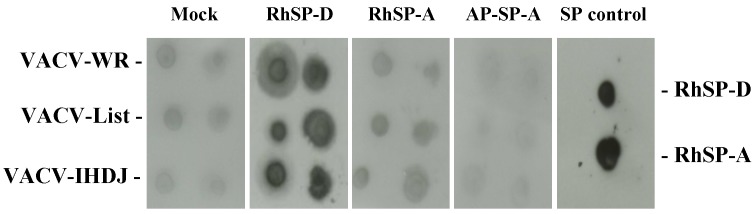
Binding of surfactant proteins to Vaccinia virus (VACV). Three VACV strains, VACV-WR, VACV-Lister and VACV-IHD-J, were dotted onto membranes and incubated with 2 µg of various surfactant proteins. After extensive washes, bound SPs were detected with specific anti-surfactant protein antibodies and revealed by enhanced chemiluminescence (ECL). Mock membrane was incubated with specific anti-SP antibodies. RhSP-D and RhSP-A, dotted onto SP control membrane, were revealed with specific anti-SP antibodies. AP-SP-A, human SP-A from alveolar proteinosis; RhSP-A, recombinant human SP-A; RhSP-D, recombinant human SP-D.

The interaction of VACV with surfactant proteins (SP) was studied using a virus-protein overlay assay (Figure 1). Solutions of purified VACV of three strains, Western-reserve, Lister and IHD-J were dotted in replicates onto nitrocellulose membranes and incubated with surfactant protein solutions. After extensive membrane washing, proteins bound to the membranes were detected using a specific anti-surfactant protein antibody (anti-SP-A and anti-SP-D) and enhanced chemiluminescence. Weak nonspecific binding was observed with the control membrane. Nonspecific signal was comparable to the signals observed with human alveolar proteinosis surfactant protein A (AP-SP-A) and human recombinant surfactant protein A (RhSP-A). RhSP-D was the only protein giving a strong interaction with VACV from different strains.

### 2.2. SP-D inhibits VACV infectivity

**Figure 2:**
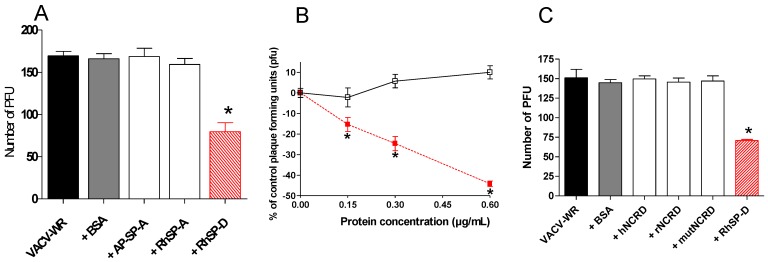
Virus infection inhibition assay. VACV-WR was incubated with proteins for 1 h. A549 cells were infected at a MOI of 0.0001 and virus replication was measured by a PFU assay. VACV-WR pre-treated groups were compared to the VACV-WR group. An asterisk indicates a statistically significant difference (p<0.05, two-tailed unpaired t-test). Incubation (A) with 0.6 µg/mL of BSA, AP-SP-A, RhSP-A or RhSP-D; (B) with increasing RhSP-D doses. Symbols: 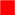, VACV-WR+RhSP-D; □, VACV-WR+BSA. (C) VACV-WR was incubated with 0.6 µg/mL of RhSP-D and 2 µg/mL of NCRD domain of SP-D from human (hNCRD), rat (rNCRD), or human E31K mutant (mutNCRD) for 1 h.

A plaque assay was used in order to determine consequences of SP-D interaction on virus infection. VACV-WR was pre-incubated with SP-A and SP-D proteins (Figure 2A). Under these conditions, the protein suspensions did not induce any cytotoxicity (data not shown). Bovine serum albumin did not modify the virus infection (p>0.05, two-tailed unpaired Student’s t-test). Pre-incubation of VACV-WR with AP-SP-A or with RhSP-A had no neutralizing activity as observed in the plaque assay (p>0.05, two-tailed unpaired Student’s t-test). By contrast, incubation of virus with SP-D resulted in strong viral neutralizing activity (53 %) when compared with non pre-incubated virus (p<0.0001, two-tailed unpaired Student’s t-test). To characterize SP-D-virus interaction, increasing concentrations of RhSP-D (0.15, 0.30 and 0.60 µg/mL) were pre-incubated with the same amount of VACV (Figure 2B). No inhibition of infection was observed with bovine serum albumin while RhSP-D induced a statistically significant dose-dependent inhibition of VACV-WR from 0.15 to 0.60 µg/mL (p=0.0033, p=0.0001 and p<0.0001, respectively; two-tailed unpaired Student’s t-test). Therefore, the activity of RhSP-D on VACV was confirmed and characterized as a dose-dependent inhibition.

### 2.3. Multimeric SP-D but not NCRD domains inhibits VACV infectivity

The inhibiting activity from full length SP-D protein was compared with trimeric NCRD domains (neck + carbohydrate recognition domain) derived from human and rat sequences (see protein structure in Scheme 1A). NCRDs were used to further characterize the requirement for multimerization of the native protein SP-D to induce viral inhibition. The human E321K mutant (mutNCRD) was previously described for its lack of HA inhibitory activity [51], resulting from the mutation of a residue involved in the coordination of calcium at the lectin site (Scheme 1C). No viral inhibition was obtained with BSA nor with any of the tested NCRD (Figure 2C) (p>0.05, two-tailed unpaired Student’s t-test) in contrast with full length SP-D protein (53% inhibition) (p<0.0001, two-tailed unpaired Student’s t-test). Additional experiments performed at 10 µg/mL of NCRD did not induce viral inhibition (data not shown). In these conditions, results suggest that the NCRD domain is not sufficient and that the multimeric state of full length protein is mandatory for efficient viral inhibition.

### 2.4. Comparison of infection inhibiting activity of SP-D with other human defense lectins

SP-D is belonging to collectins which are oligomeric proteins consisting of collagen stalks and globular carbohydrate recognition domains. The other collectin MBL and the related proteins L-, H- and M-ficolins were tested for viral inhibition in comparison to SP-D (Supplementary data 1). Equal quantities of proteins were tested in this experiment. SP-D was the only collectin leading to VACV-WR infection inhibition (about 50% of inhibition) (p<0.0001, two-tailed unpaired Student’s t-test). 

### 2.5. Recombinant A27 viral protein binds to SP-D

**Figure 3:**
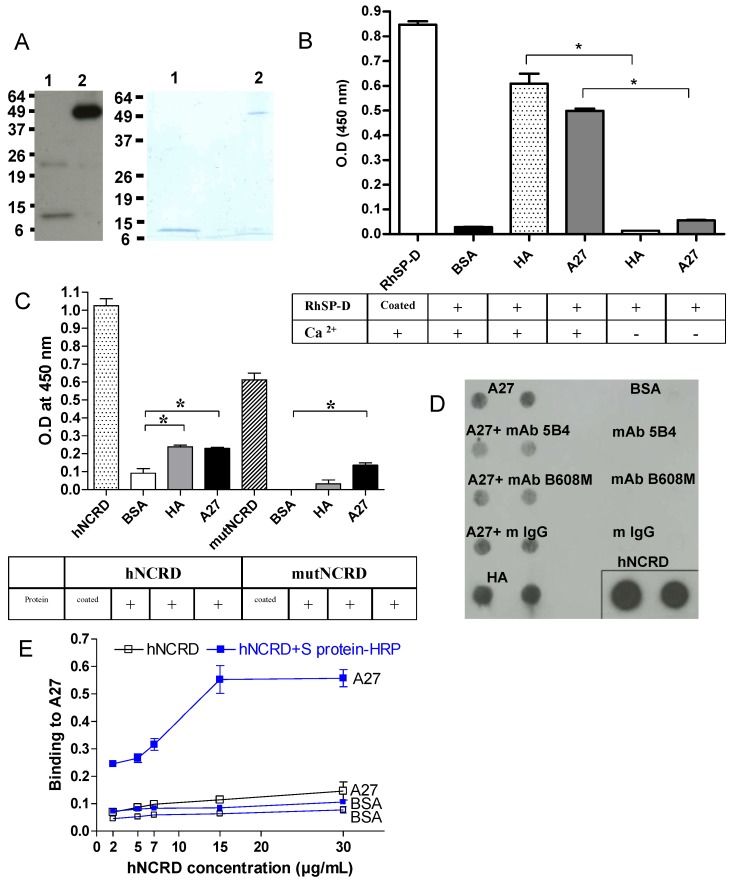
A27 viral protein interacts with SP-D. (A) Characterization of the recombinant A27 protein. The purified recombinant A27 protein (0.5 µg/lane and 2 µg/lane for Western-blot (left panel) and 12% SDS-PAGE analysis (right panel), respectively) was heated at 100°C for 5 min in denaturating, reducing sample buffer (lane 1) or incubated for 5 min at room temperature in non-reducing sample buffer (lane 2). RhSP-D (2.5 µg/ml) (B) or hNCRD and mutNCRD (5 µg/ml) (C) were incubated for 1 hour at 37°C with coated proteins on 96-multiwell plate. Bound RhSP-D was revealed with a rabbit anti-SP-D specific antibody and with a goat anti-rabbit-HRP antibody. TMB substrate was added and OD was measured at 450 nm. An asterisk indicates a statistically significant difference (n=3; p<0.05, two-tailed unpaired t-test). (D) Interaction between A27 and hNCRD was partially inhibited with mAbs against A27. A27 was preincubated with mAbs at 10 µg/mL. 1 µg of A27 protein was dotted onto membrane and incubated with hNCRD (5 µg/mL). 0.5 µg of hNCRD was dotted onto the membrane as positive detection control. After extensive washes, bound hNCRD was detected with specific S protein-HRP antibody and revealed by enhanced chemiluminescence (ECL). (E) Both A27 protein and BSA were coated on 96-multiwell plate. A range (2, 5, 7, 15 and 30 µg/mL) of hNCRD alone or hNCRD preincubated with S protein-HRP (1:20, concentration) was incubated for 1 hour at 37°C. Preincubation of hNCRD with S protein-HRP noticeably increased binding of hNCRD to A27. BSA, bovine serum albumin; HA, hemagglutinin of IAV; A27, A27 viral protein of VACV (14-kDa fusion protein). Incubation was performed in the absence (-) or presence (+) of 5 mM Ca ^2+^.

The binding of A27 protein to SP-D was investigated *in vitro* using an ELISA assay. A27 viral protein is a membrane protein localized on the surface of the IMV, the most abundant virus form produced in infected cells known to be responsible for the initiation of infection. An *E. coli* expression system was used because A27 protein produced in this system was shown to exhibit structural and functional properties similar to those found in the virus particle and in virus-infected-cells [52]. To determine the involvement of the A27 viral protein in SP-D interaction, A27 was expressed in a soluble form in *E. coli*, and purified by nickel affinity chromatography using the hexahistidine tag at the N-terminus of the recombinant protein. As determined by SDS-PAGE analysis, in denaturing and reducing conditions A27 migrated as one band (14 kDa, monomer) detected by Coomassie blue staining and as two bands (an additional minor band at 20 kDa) that was detected by western-blotting (Figure 3A, lane 1). In non-reducing conditions, A27 spontaneously assembled into trimers (Figure 3A, lane 2). Despite the same amount of protein applied in each lane, a difference in A27 band intensities (lanes 1 and 2) was observed which may result from the weaker capacity of the monoclonal antibody to react with the monomeric state of A27. N-glycosidase F digestion experiments confirmed the expected absence of sugars (data not shown). 

The interaction of SP-D with A27 was examined onto microtiter wells (Figure 3B). Coated proteins were incubated with RhSP-D. Bound RhSP-D was detected with a specific antibody against SP-D. A mix of HA of IAV was used as a positive control of interaction. RhSP-D interacted with A27 in presence of Ca ^2+^ whereas the binding was abolished for both HA and A27 proteins, in the absence of Ca ^2+^. The experiment indicated that the binding of SP-D to A27 is Ca^2+^-dependent. 

Contribution of NCRD domains in the interaction between SP-D and A27 was examined in microtiter wells (Figure 3C). Coated proteins were incubated with hNCRD or mutNCRD in presence of Ca ^2+^. Bound NCRDs were detected with a specific antibody against SP-D. A mix of HA of IAV was used as a positive control of interaction. A weak interaction was obtained for hNCRD with both A27 and HA whereas no binding of mutNCRD to HA was observed. The experiment suggested that NCRD of SP-D could contribute to the interaction with A27. The weak interaction of hNCRD with A27 was maintained with the mutNCRD, suggesting the region of the CRD near to, but distinct from, the lectin site contributed to the interaction.

We next studied whether monoclonal antibodies (mAbs) directed against the A27 protein of VACV could prevent the interaction between A27 and hNCRD, using a protein-protein overlay assay (Figure 3D). A27 solutions were dotted in replicates onto nitrocellulose membranes and incubated with hNCRD. After extensive membrane washing, bound hNCRD was detected using S protein-HRP antibody (anti-His antibody was avoided as both hNCRD and A27 were His-tagged proteins) and enhanced chemiluminescence. No binding was observed with the control spots (mAbs alone). A weak specific signal was observed with A27 and hNCRD compared to HA of IAV and hNCRD. No inhibition was observed with control mouse IgG while mAb against A27 (5B4) partially inhibited the A27-hNCRD interaction.

To clarify the finding obtained with NCRD, a higher oligomeric structure of NCRD was tested using the capacity of the multivalent S protein-HRP to generate aggregates of NCRD containing the S protein binding site [53] [28]. In fact, NCRD contains an S protein binding site, which can be detected by the S protein conjugated with HRP and subsequently used to detect the binding to various ligands. Preincubation of hNCRD with the S protein-HRP complex resulted in considerably increased binding to A27 compared to the situation where hNCRD is added to the viral protein followed by addition of S-protein-HRP (Figure 3E). A similar result was obtained with HA (data not shown), thus indicating that multivalency of NCRD is required to potentiate the interaction with A27.

### 2.6. Visualizationof the interaction of SP-D to VACV particle by electron microscopy (EM)

**Figure 4:**
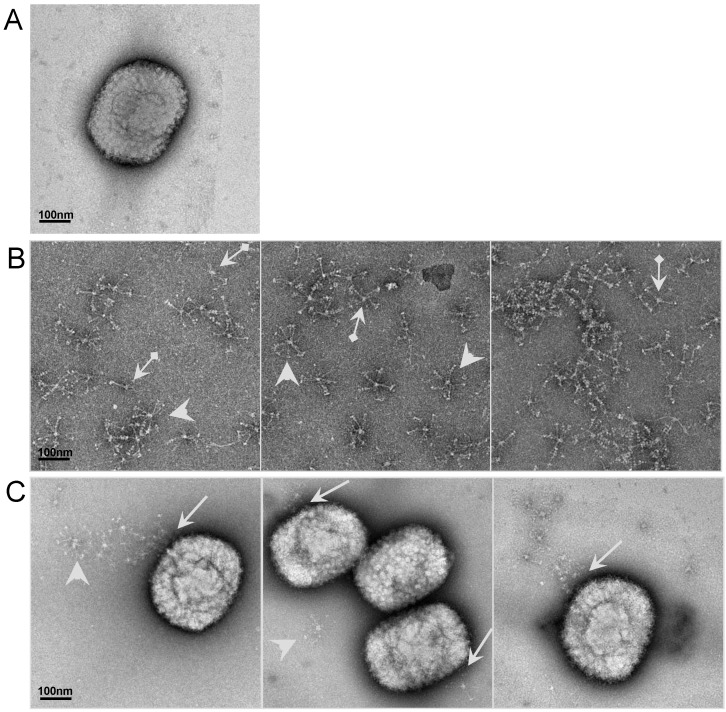
Electron Microscopy of virus particle interacting with SP-D. The interaction between inactivated VACV-WR and RhSP-D was illustrated. Panel A represents the inactivated virus. Panel B represents the control of RhSP-D (arrows: dodecamer, cruciform structure; and arrow heads: higher order multimers). Panel C displays the interaction and the site of attachment (arrows) between VACV-WR and RhSP-D; arrow heads represent isolated protein. The bar corresponds to 100 nm.

To further study the interaction between SP-D and VACV viruses, electron microscopy was performed on inactivated VACV-WR incubated with RhSP-D (Figure 4). After negative staining, the VACV particles were observed by EM (Figure 4A). The electron micrographs showed the polymeric forms of RhSP-D as previously described for both bronchoalveolar lavage purified SP-D and RhSP-D as “astral bodies” [54,55]. Preparation contained only some dodecamers (cruciform structures) and a majority of higher order multimers of RhSP-D (Figure 4B). The globular heads of SP-D, containing the CRD, can be seen especially on the periphery of the radially symmetric multimers. In presence of the virus particle, no interaction was observed with SP-A (data not shown) whereas SP-D interacted with the surface of the virus particle, which binds at one part of the virus particle (Figure 4C). 

### 2.7. Analysis of SP-D binding to VACV by surface plasmon resonance (SPR) spectroscopy

#### 2.7.1. SP-D binding to viral particle

**Figure 5:**
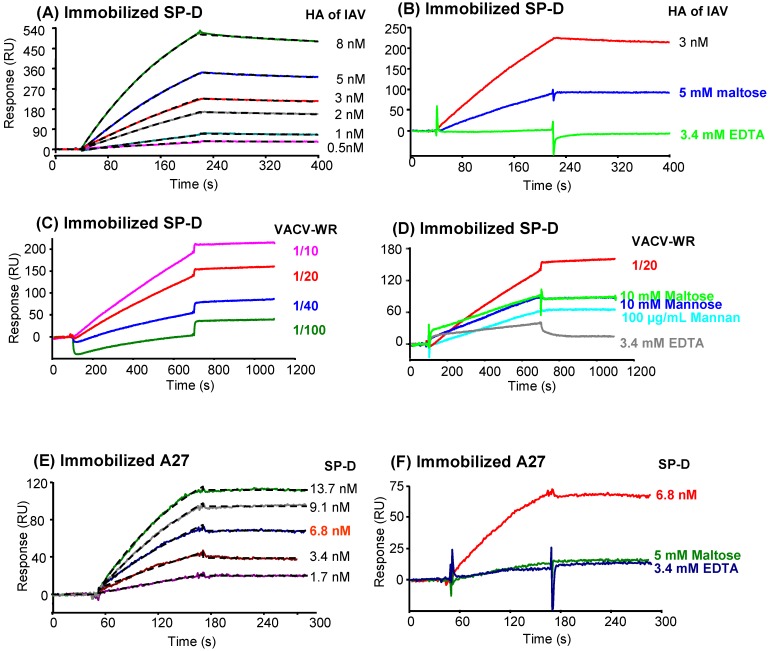
SPR analysis of the interaction of VACV with immobilized SP-D. *VACV-WR particle binding to SP-D:* (A) SPR analysis of the interaction of HA with immobilized SP-D. HA of IAV was injected at concentrations of 0.5, 1, 2, 3, 5 and 8 nM over immobilized SP-D. (B) HA (3 nM) was injected over immobilized SP-D in the presence of selected competitors of SP-D-ligand interaction (5 mM maltose or 3.4 mM EDTA). (C) iVACV was injected at various concentrations ranging from equivalent titer 2.10^8^ to 2.10^9^ virus/mL (corresponding to 1/100 to 1/10 dilutions, respectively) over immobilized SP-D. (D) iVACV (1/20 dilution) was injected over immobilized SP-D in the presence of competitors of SP-D interaction with carbohydrates (10 mM maltose, 10 mM mannose, 100 µg/mL mannan or 3.4 mM EDTA). All results are representative of two independent experiments. Sensorgrams were distorted at the beginning and end of injections due to solvent effect. *Binding of SP-D to viral A27 protein*: (E) Purified SP-D was injected at concentrations of 1.7, 3.4, 6.8, 9.1 and 13.7 nM over immobilized A27. (F) SP-D (6.8 nM) was injected over immobilized A27 in the presence of 5 mM maltose or 3.4 mM EDTA. Results are representative of two independent experiments.

SPR spectroscopy was used to further characterize the interaction of SP-D with VACV. First, the well characterized interaction between HA of IAV and SP-D was verified by SPR, allowing for confirmation of previous studies using other techniques [23,31,47]. Soluble HA bound in a dose-dependent manner to immobilized SP-D in the presence of calcium ions (Figure 5A). Kinetic analysis of these binding data yielded association and dissociation rate constants of 0.7 x 10^5^ M^-1^ s^-1 ^and 1.2 x 10^-4^ s^-1^ respectively, using global fitting to a 1:1 Langmuir interaction model. The deduced apparent equilibrium dissociation constant (*K*_D_) was 0.53 nM, indicative of high affinity. However, due to the oligomeric nature of both SP-D and HA, it cannot be excluded that some rebinding might occur during the dissociation phase, thus enhancing the apparent stability of the complex. Inhibition of the interaction between HA and SP-D was obtained using maltose and EDTA (Figure 5B). 

Injection of various concentrations of iVACV over immobilized SP-D (Figure 5C), yielded dose-dependent responses ranging from 50 to 200 resonance units using equivalent titers of iVACV ranging from 2.10^8^ to 2.10^9^ virus /mL (corresponding to 1/100 and 1/10 dilutions, respectively). The affinity of the interaction could not be quantified since determination of the dissociation constant requires precise knowledge of the molar concentration of the soluble interactant, which does not apply to a viral particle with many surface-exposed potential ligands. Using a fixed viral dose (D=1.10^9^ equivalent titer of inactivated virus /mL), different potential inhibitors were tested (Figure 5D). Inhibitors of specific CRD attachment, maltose, mannose and mannan induced 32%, 36% and 53% of inhibition at this specific dose, respectively. The calcium chelator induced a strong inhibition of 78% (Figure 5D). These results showed the calcium-dependence of the interaction between VACV and SP-D and the inhibitory effect of sugar ligands of SP-D, thus strongly suggesting the involvement of the carbohydrate-binding domain of the collectin.

#### 2.7.2. Characterization of the interaction between SP-D and recombinant A27

HA of IAV was demonstrated to strongly interact with SP-D (see Figure 5A), moreover it was shown to present common domains and function with A27 vaccinia virus protein [35]. To determine A27 protein involvement in SP-D interaction with VACV, trimeric recombinant A27 was immobilized on a sensor chip and binding of SP-D to A27L analyzed using SPR spectroscopy (see Figure 5E). SP-D bound to A27L in a dose-dependent manner and the dissociation was very slow, reflecting the great stability of the complex (Figure 5E). Determination of the kinetic parameters yielded *k*_a_ and *k*_d_ values of 5.8 x 10^5^ M^-1^ s^-1^ and 8.9 x 10^-5^ s^-1^, with a resulting apparent *K*_D_ value of 0.15 nM, indicative of strong affinity. Again, these values are only apparent due to the multivalent nature of the interaction, but they are in the same range as those obtained for the interaction of HA with SP-D under the same conditions. Inhibition of interaction was also observed in the presence of maltose and EDTA (Figure 5F).

#### 2.7.3. Characterization of the interaction of A27 with other proteins of the defense collagen family

The capacity of other proteins of the defense collagens family to interact with A27L was tested by monitoring binding of the proteins to immobilized A27 using SPR spectroscopy (Supplementary data 1B). As shown in the upper panel, all proteins tested bound to A27, although clearly to different extents: the binding level for MBL was in the same range as that of SP-D, whereas the three human ficolins, especially M- and H-ficolins, exhibited poor A27 binding capacity. We decided next to determine if the NCRD domain from SP-D was sufficient to mediate interaction with A27 (Supplementary data 1B, lower panel). Human NCRD (hNCRD) and E321K, a mutant NCRD (mutNCRD) with impaired binding capacity to a variety of microorganisms recognized by SP-D [50] showed a weak interaction with A27. When using a 90 nM concentration of hNCRD, a weak interaction was detected. However, similar results were obtained at a concentration of 900 nM thus casting doubts about the specificity of the observed interaction (data not shown). Both SP-A (RhSP-A and AP-SP-A) showed no interaction with A27 (lower panel). Among the tested lectins, SP-D showed the strongest interaction with A27. Because trimeric NCRD was inactive, the data suggest important cooperative interactions among CRDs.

### 2.8. Protective role of SP-D in VACV infection in mice model

**Figure 6:**
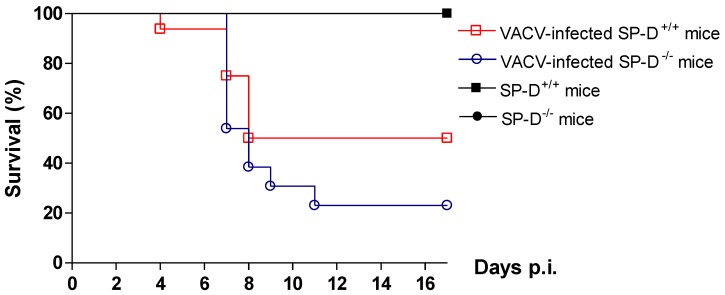
SP-D protected mice against VACV-WR. Five week old Black Swiss mice SP-D^+/+^ and SP-D^-/-^ were infected intranasally with 1 LD_50_ of VACV-WR. Survival was monitored for 17 days. SP-D^-/-^ group was compared to SP-D^+/+^ group and to a mock-infected group (Log Rank test).

To address the role of SP-D in VACV susceptibility *in vivo*, SP-D^+/+^ and SP-D^-/-^ (deficient in SP-D) were infected with VACV-WR by intranasally instillation. The VACV-WR strain, shown to bind SP-D *in vitro*, was chosen because of its ability to induce death in mice [56]. LD_50_ calculated on SP-D^+/+^ mice was estimated at 5 x 10^5^ PFU (data not shown). The experiment was performed three times with comparable and significant results, one representative experiment being shown (Figure 6). At 1 LD_50_ inoculating dose, VACV-WR was responsible for about 50% death of SP-D^+/+^ mice whereas all uninfected animals survived. Difference in survival was observed between VACV-infected SP-D^+/+^ and VACV-infected SP-D^-/-^ mice groups. In the absence of SP-D, the pathogenesis of VACV was increased, only 23% infected SP-D^-/-^ mice survived compared to 50% in the infected SP-D^+/+^ mice group (p=0.0069; Log Rank test). 

The importance of the lung as a primary target during respiratory transmission of variola virus highlighted the importance to study influence of surfactant during infection [42]. It is likely that lung surfactant defense proteins play a role in containing the beginning of the infection [23]. Surfactant innate defense system is mainly composed of collectins and phagocytes. Collectins or C-type lectins are composed of polypeptides including mannan-binding lectin, bovine conglutinin and two surfactant proteins: SP-A and SP-D. This family shares structural features such as a collagenous N-terminal domain, a neck and a globular C-terminal domain which is implicated in carbohydrate recognition leading to pathogens opsonization and clearance [57].

This study provides the first evidence for an inhibitory effect of SP-D on poxvirus infectivity *in vitro* and its exclusive activity compared to other collectins. Among pulmonary collectins, SP-D was the only protein leading to a strong interaction with VACV in our study. Three strains of VACV were tested (Western Reserve, Lister and IHD-J) and no difference was observed. SP-A (recombinant and alveolar proteinosis purified forms) was unable to interact with any viral strain contrary to SP-D, which strongly interacted with all strains in a similar manner. These results demonstrated a specificity of binding between SP-D and VACV contrary to Influenza A Virus (IAV) that interacts with both SP-A and SP-D [47,58].

Using a pulmonary cell line, this interaction was directly linked to the ability of SP-D to inhibit virus infection when using a pre-incubation plaque assay. Implication of Neck + Carbohydrate Recognition Domain (NCRD) was tested but no inhibition was obtained with any of the two recombinant NCRD domains (human and rat). This result indicates that purified NCRD alone is not sufficient but may require higher level of organization (multimerization) for being active or that full length protein is needed for inhibition activity. A similar result was previously described for IAV, with native SP-D being protective *in vivo* but not wild-type NCRD alone [53] [28]; however, certain mutant NCRDs with greatly enhanced IAV binding activity did show antiviral activity *in vivo* [59,60].

Influenza A virus is one of the most extensively respiratory virus studied. Its interaction with SP-D was widely studied and authors agreed that HA mediated-interaction is sugar inhibited and Ca-dependent, resulting from the involvement of CRD. While we could confirm these observations between for HA of IAV by ELISA and SPR spectroscopy, we could not completely elucidate the mechanism of interaction between A27 and SP-D. SPR spectroscopy experiments showed that SP-D was able to interact with VACV in a dose-dependent manner and that the carbohydrate recognition domain (CRD) should be implicated because of binding inhibition by sugars and EDTA. In the same manner, a Ca^2+^-dependent interaction was first observed between SP-D and A27 coated on microtiter plates. The lack of neutralization of virus infection in plaque assay by NCRD and the weak interaction observed between A27 and hNCRD suggested that the involvement of NCRD domain alone (trimeric form) is not sufficient whereas the multimerization of NCRD (higher oligomeric form using S protein-HRP) resulted in a marked interaction. The involvement of multimerized NCRD domains could not be confirmed by SPR due to difficulties of interpretation of the data (presence of glycerol in S protein-HRP).

Although it appears clear that efficient binding requires oligomerization of the CRDs, the mechanism of SP-D interaction with A27 was not entirely clarified. On the one hand, the fact that the binding is inhibited by calcium chelation and in the presence of maltose suggests an involvement of the lectin activity of SP-D. On the other hand, the fact that the weak interaction with NCRD was maintained with the E321K mutant devoid of lectin activity and that the A27 protein is not glycosylated favors the hypothesis of a protein-protein interaction. The interaction of SP-D with A27 appears thus different from that of SP-D with HA, known to involve N-linked carbohydrates of the viral protein [61]. It is rather reminiscent of previous data on the interaction of SP-D with the extracellular domain of TLR-2 and -4, showing that N-linked oligosaccharides are not required and suggesting an involvement of amino acid residues close to the ligand binding site of SP-D [19]. Alternatively, binding of the CRD to a ligand such as maltose may induce conformational changes and impair subsequent interaction with A27. Anti-SP-D monoclonal antibodies would be helpful to further identify the SP-D sequence that supports binding to A27 [19]. In the same manner, a mutant of the multimeric SP-D protein with a non-functional NCRD (e.i E321K mutation) would be useful to determine which domain is important for binding to A27. 

Among surfactant proteins, SP-D was the only protein able to inhibit viral infection when using a pulmonary cell line and the soluble defense collagens tested (SP-A, MBL and the three human ficolins (H, L and M)) were unable to induce inhibition. However, SPR experiments showed that some of these proteins interacted with A27 (MBL, L-ficolin and M-ficolin). This apparent discrepancy is reminiscent of previous observations of MBL interaction with Ebola virus. Even if MBL could bind, interact and block Ebola envelope glycoprotein-mediated virus binding to DC-SIGN, MBL was unable to directly neutralize the virus without the presence of complement in neutralization assays [62]. In the same manner, neutralization of influenza virus by MBL was complement-dependent [63]. Little information is available concerning the interactions between ficolins and viruses. In contrast, neutralization of viruses by SDP-D was observed in the absence of complement, conditions in which our experiments were performed. It should be mentioned that, contrary to SP-D, interaction of MBL and ficolins with viruses is expected to activate the lectin complement pathway, leading to opsonization of the virus by C3b fragments for subsequent phagocytosis.

Inhibition of VACV in an epithelial airway cellular model led us to use a SP-D-deficient mice model. The mortality of VACV-infected mice was increased in the absence of SP-D, suggesting the involvement of SP-D to neutralize VACV infection. Future experiments could be performed by measuring virus titers in lung, cells recruitment [48] [64], pro-inflammatory cytokine production [49] [48] and histopathological changes to explain the difference of mortality between SP-D ^-/-^ and wild-type mice and to address the significance of SP-D in protection against VACV infection.

## 3. Experimental Section

### 3.1. Cells and viruses

A549 cells (human lung carcinoma cell line, ATCC CCL-185) were grown in F12K medium (Gibco). BHK-21 cells (hamster kidney cells, ATCC CCL 10) were grown in Glasgow minimum essential medium (Gibco) supplemented with 10% tryptose phosphate (Sigma) and 50 mM HEPES (Gibco). Vero cells (African green monkey kidney cells, ATCC CCL 81) were grown in M199 medium (Gibco). For cell culture, all media were supplemented with 10% inactivated fetal calf serum (FCS), whereas medium for infection was supplemented with 5% FCS and 1% antibiotics (100 U/mL of penicillin and 100 µg/mL of streptomycin, Gibco). Cells were cultured at 37°C in a 5% CO_2_ atmosphere. Cytotoxicity was determined by trypan blue staining and cell counting. The vaccinia virus Western Reserve strain (VACV-WR) was obtained from the ATCC (ATCC VR-119), the first-generation Lister smallpox vaccine (VACV-List, X55-33) was provided by the French health authorities and the vaccinia virus IHD-J strain (VACV-IHDJ) was obtained from the ATCC (ATCC VR-156). All virus strains were produced in BHK-21 cells, intracellular mature viruses were purified using sucrose density gradient centrifugation as previously described [65]. Virus was stored in 10 mM Tris-HCl (pH 8) at -80°C. Virus productions were titrated in Vero and A549 cells. Before infection, cells were rinsed and infected with virus in culture medium supplemented with 0.5% FCS and 1% antibiotics. Purified viruses were inactivated (iVACV) for some experiments using a previously described protocol [66]. Briefly, 100 µL were mixed with 1 µL Psoralene solution (Sigma, 1mg/mL in DMSO) and exposed during 1 hour to an UV light (365 nm) in a tissue culture plate 48 multi-wells plate. Equivalent titer of iVACV was 2 x 10^10^ plaque forming units (PFU)/mL.

### 3.2. Reagents

Rabbit anti-surfactant antibodies raised against SP-A and SP-D proteins were purchased from Millipore (AB3420 and AB3434, respectively). Rabbit anti-vaccinia whole virus antibody was purchased from Fitzgerald-Interchim (20VR-69) and both peroxydase-conjugated goat anti-rabbit and anti-mouse IgG antibodies were purchased from Jackson ImmunoResearch. Mouse monoclonal anti-His antibody was purchased from Qiagen (34 660). The mouse monoclonal anti-A27 antibody (5B4) was obtained from C.P Czerny and the mouse monoclonal anti-VACV antibody (B608M) was purchased from Abcam (Ab48569). Peptide:N-glycosidase F (PNGase F) was purchased from Sigma (P7367). A mix of recombinant HA proteins of IAV were obtained from Vaxigrip® (A/Brisbane/59/2007 (H1N1); A/Brisbane/10/2007 (H3N2); B/Florida/4/2006). Predicted molecular size of HA (trimeric protein) was 180 kDa (extracellular domain). S protein-HRP conjugate was purchased from EMD Chemicals, Inc (69047-3).

### 3.3. Recombinant surfactant proteins

Recombinant human SP-D dodecamers were expressed in CHO-K1 cells and purified as previously described [55]. Recombinant trimeric neck + carbohydrate recognition domain fusion proteins (NCRDs) from human (hNCRD and the E321K mutant (mutNCRD)) and rat (rNCRD) species were expressed in bacteria and purified as previously described [57] [50]. All preparations used for these studies had low endotoxin (45.2 ng LPS/mg of RhSP-D protein; 20 ng LPS/mg of hNCRD; 0.56 ng LPS/mg of rNCRD; 0.27 ng LPS/mg of mutNCRD). AP-SP-A was a kind gift from Dr J.R. Wright (Duke University, Durham, USA). Recombinant RhSP-A was kindly provided by Dr F. McCormack (University of Cincinnati, Cincinnati, USA). The molecular size of proteins was estimated as followed: 516 kDa for RhSP-D (composed of twelve identical polypeptides of 43 kDa) and 72 kDa for both hNCRD and mutNCRD (composed of three identical CRD domain of 24 kDa).

### 3.4. Other recombinant proteins

Human recombinant wild-type M-ficolin was expressed in S2 insect cells and purified as described previously [67]. L-ficolin was purified from serum as described previously [68]. Recombinant human MBL and H-ficolin were produced in CHO cells and purified as described by Teillet *et al.* [69] and Lacroix *et al.* [70], respectively.

### 3.5. A27 viral protein expression and purification

The gene coding for full length A27 protein (*A27L*) was amplified from Lister strain of VACV (Pourquier vaccine) using the following primers: A27 sense primer: 5’GGGGACAAGTTTGTACAAAAAsAGCAGGCTTAATGGACGGAACTCTTTT3’ and A27 antisense primer: 5’GGGGACCACTTTGTACAAGAAAGCTGGGTTTTACTCATATGGACGCCG3’. *A27L* construct was finally cloned in pDEST^TM^17 (Invitrogen, Gateway technology) using the following primers: attB1-A27LS : 5’GGGGACAAGTTTGTACAAAAAAGCAGGCTTAATGGACGGAACTCTTTT 3’ et attB2-A27LR : 5’GGGGACCACTTTGTACAAGAAAGCTGGGTTTTACTCATATGGACGCCG 3’ and transformed in BL21 (DE3) pLysS. The recombinant A27 protein (rA27) contained an N-terminal His-tag by analogy with previous studies showing that an N-terminal T7 tag peptide did not interfere with the function of the corresponding A27 construct [36,37,38]. Colonies were used to inoculate 1 L of LB containing appropriate antibiotics. Cells were grown in shakers at appropriate temperature (37°C) and speed (200 rpm) until reaching 0.6 OD at 600 nm. Expression was induced with 1mM final concentration of IPTG for 4.5 hours at 37°C. Pellets were harvested after centrifugation (10000g, 5 min, 4°C). They were resuspended in lysis buffer (Tris-HCl 1.5 M, pH 7.3), then sonicated and supernatant was harvested. Recombinant N-terminal-His tagged protein was loaded on a His Trap^TM^ HP column (Amersham Biosciences) and eluted using buffer containing 20 mM sodium phosphate, 500 mM NaCl and 500 mM imidazole. Purified protein was dialyzed overnight against PBS and quantified using Bradford assay. 240 µg of purified rA27 (Quick Start Bradford Protein Assay) was obtained from 1 L of induced bacteria. The expected amino acid sequence of rA27 was MSYYHHHHHHLESTSLYKKAGLMDGTLFPGDDDLAIPATEFFSTKAAKKPEAKREAIVKADEDDNEETLKQRLTNLEKKITNVTTKFEQIEKCCKRNDEVLFRLENHAETLRAAMISLAKKIDVQTGRRPYE, underlined residues corresponding to A27. The monomeric and trimeric forms of the A27 protein were detected by electrophoresis on a 12% SDS-polyacrylamide gel under reducing and non-reducing conditions, respectively, and protein profiles revealed by western-blot analysis and ECL detection and autoradiography using a mouse monoclonal anti-His antibody.

### 3.6. A27 SDS-PAGE and western-blot analyses

A27 recombinant protein was subjected to SDS-PAGE (12%) under reducing and non-reducing conditions. Using western-blot method, A27 protein was revealed using an anti His antibody and a HRP-labeled goat anti-mouse IgG antibody. The peroxidase reaction was assessed by using ECL. The purified recombinant A27 protein (0.5 µg) was digested by 15 U peptide:N-glycosidase F (PNGase F) for 10 hours at 26°C.

### 3.7. Surfactant protein interaction with VACV strains by overlay assay

Purified mature viruses of VACV-WR, VACV-List and VACV-IHDJ strains were dotted on membranes. Duplicates of viral solutions (2 µg/spot, Bradford virus titration) were spotted onto HybondC-extra nitrocellulose membranes (Amersham) and allowed to dry in the dark at room temperature (RT) for one hour. Membranes were blocked for one hour at RT in TBS containing 0.5% Tween 20, CaCl_2_ 5 mM and 3% fatty acid-free BSA (Sigma). The membranes were then incubated overnight at RT in the same buffer containing 1 µg/mL surfactant proteins. The membranes were washed three times for 10 min and then incubated for 1 h at room temperature with the rabbit anti-SP-A or anti-SP-D antibody (1/1000 containing 3% BSA). After four 20-min washes, the membranes were incubated for 1 h with anti-rabbit horseradish peroxidase conjugate (1/10000 dilution). Finally, after six 10-min washes, the interactions were detected by enhanced chemiluminescence (ECL). 

### 3.8. hNCRD interaction with A27 by overlay assay

Purified A27 and HA (1 µg/spot) were dotted onto HybondC-extra nitrocellulose membranes (Amersham). Membranes were blocked for 1 hour at RT in TBS containing 0.05% Tween 20, CaCl_2_ 10 mM and 5% milk. A27 was preincubated with mAbs (clone 5B4, Abcam (B608M) and mouse Ig) for 30 minutes at 37°C at 10 µg/mL. The membrane was then incubated overnight at 4°C in the same buffer containing 5 µg/mL hNCRD. The membranes were washed three times for 20 min and then incubated for 1 h at room temperature with the S protein-HRP antibody (1/10,000). After three 20-min washes, interaction was detected by enhanced chemiluminescence (ECL). 

### 3.9. Lectins-virus infection inhibition assay

A549 cells were seeded to obtain confluent plates after 48 hours of culture in 12 multi-wells plates. VACV-WR was incubated with 0.6 µg of SP-D, MBL, ficolins or 2 µg of human, rat and mutated E321K human NCRD for 1 hour at 37°C. During this time, cells were rinsed with fresh F12K medium 0.5% FCS and 1% antibiotics. Then, virus-protein mixes were incubated with cell monolayer (multiplicity of infection, MOI 0.0001) at 37°C in 5% CO_2_ atmosphere during one hour. Cells were rinsed with 0.5% FCS medium and covered with 1.5 mL fresh F12K medium 5% FCS, and cultured for an additional 48 hours. Cells were fixed by adding 0.75 mL of fixing and staining solution (0.2% crystal violet, 4.5% formaldehyde and 7.5% ethanol in PBS) per well for 2 hours. Wells were rinsed twice with water and PFU were counted. Experiment was performed three times. Additional experiment was performed at 10 µg of human NCRD.

### 3.10. Binding of SP-D to A27 recombinant VACV protein

A27, HA mix of IAV (Vaxigrip^®^), SP-D, hNCRD and mutNCRD (100 ng/well in 100 mM Na_2_CO_3_ pH 9.6) were coated onto microtiter wells overnight at 4°C, and nonspecific binding was blocked with PBS buffer without Ca and Mg (BioWhittaker) containing 5% (w/v) fatty acid-free BSA, 0.05% tween and 5 mM CaCl_2_ (buffer A) for 1 hour at 37°C. SP-D (2.5 µg/mL) or NCRD (5 µg/mL) in buffer A was added and incubated at 37°C for 2 hours. After the incubation, the wells were washed with buffer A and further incubated with rabbit anti-SP-D polyclonal antibodies followed by incubation with peroxidase-conjugated goat anti-rabbit IgG antibody. The peroxidase reaction was finally conducted by using TMB substrate (Sigma), and the absorbance at 450 nm was measured. Some incubations were also performed in the absence of Ca^2+^.

### 3.11. ELISA assay for measurement of binding of NCRDs to A27 recombinant VACV protein

Binding of trimeric NCRD fusion proteins to A27 protein of VACV was measured as previously described using the S protein-HRP conjugate (Novagen) [53]. In brief, ELISA plates were coated with A27 protein (10 µg/mL, 50 µL per well) followed by washing and addition of a range (2, 5, 7, 15 and 30 µg/mL) of NCRD alone or NCRD that had been preincubated with S protein-HRP (1:20, concentration). After further washing, S protein-HRP was added to the wells that had only received NCRD (1:5 000, concentration). Binding was detected using peroxidase substrate (TMB, Sigma, France).

### 3.12. Electron Microscopy

Inactivated VACV-WR was incubated with RhSP-D for 1 h at 37°C (vol/vol). Inactivated VACV-WR and RhSP-D were used as a control. After incubation 3 µl of each sample was adsorbed to a carbon-mica interface. The carbon film with adsorbed protein was floated onto a solution of a 2% uranyl acetate solution diluted in water. The film was then picked up by a 200 mesh copper EM grid and finally air-dried. The samples were then transferred in an FEI Tecnai electron microscope equipped with a CCD cold stage 2x2K camera and observed at 200 Kv. 

### 3.13. Surface plasmon resonance analyses on immobilized SP-D

Analyses were performed using a Biacore X instrument (GE Healthcare). Recombinant human SP-D and fatty acid-free BSA were diluted to 23 µg/ml in 10 mM Na acetate, pH 5.0 and 50 µg/ml in 10 mM Na acetate, pH 4.0, respectively, and immobilized on the surface of a CM5 sensor chip (GE Healthcare) using the amine coupling chemistry until coupling levels of 10,000 RU (SP-D) and 8,800 RU (BSA) were reached. The running buffer for immobilization was 10 mM Hepes, 150 mM NaCl, 3.4 mM EDTA, pH 7.4 containing 0.005% surfactant P20 (GE Healthcare). Fifty µl of inactivated WR virus were injected over both SP-D and the blank surface at a flow rate of 5 µl/min in 20 mM Hepes, 150 mM NaCl, 1 mM CaCl_2_, pH 7.4, containing 0.005% surfactant P20. Surfaces were regenerated with 10 µl of 10 mM EDTA, pH 7.4, with an additional injection of 1 M NaCl, 10 mM EDTA, pH 7.4, when needed. The binding curves shown are obtained after automatic subtraction of the signal recorded on the reference surface. Injections (40 µl) of various concentrations of a mix of IAV hemagglutinin (Vaxigrip®) for kinetic analysis were performed under the same conditions, except that the flow rate was set at 20 µl/min. Experiments were performed twice.

### 3.14. Surface plasmon resonance analyses on immobilized A27 viral protein

Recombinant viral A27 and fatty acid-free BSA were diluted to 26 and 20 µg/ml, respectively, in 10 mM Na acetate, pH 3.5, respectively and immobilized on the surface of a CM5 sensor chip using the amine coupling chemistry until coupling levels of 600 RU (A27) and 1400 RU (BSA) were reached. Forty µl of various lectins (SP-D, SP-A, MBL, ficolins) were injected over both A27 and the blank surface at a flow rate of 20 µl/min in 20 mM Hepes, 150 mM NaCl, 1 mM CaCl_2,_ pH 7.4, containing 0.005% surfactant P20 (GE Healthcare). Surfaces were regenerated with 10 µl of 1 M NaCl, 10 mM EDTA, pH 7.4, with an additional injection of 0.1% SDS, when needed. The binding curves shown were obtained after automatic subtraction of the signal recorded on the reference surface. Experiments were performed twice.

### 3.15. Surface Plasmon resonance data evaluation

Kinetic data were analyzed by global fitting to a 1:1 Langmuir binding model of both the association and dissociation phases for at least five concentrations simultaneously (1.7-13.7 nM SP-D, 0.5-8 nM hemagglutinin of IAV), using the BIAevaluation 3.2 software (GE Healthcare). The apparent equilibrium dissociation constants (*K*_D_) were calculated from the *ratio* of the dissociation and association rate constants (*k*_d_/*k*_a_).

### 3.16. Ethics Statement

Animals were housed according with the French Ethical Committee (Decree 87-848) and European Community Directive 86/609/EEC. Experiments were carried out under the supervision of the “Comité Consultatif pour l’Éthique en Expérimentation Animale”. The protocols were approved by the Committee on the Ethics of Animal Experiments of the IRBA-CRSSA (Permit Number 2007/33.01). All efforts were made to minimize suffering.

### 3.17. Animals

National Institutes of Health (NIH) Swiss Black SP-D^+/+^ and knocked out SP-D^-/-^ mice (females and males) were provided from Cincinnati Children’s Hospital Medical Center (Ohio, USA) [71] and finally bred in Institute Jean Roget animal facility (La Tronche, France). Mice (four weeks old) were quarantined for one week. General anesthesia was performed by intraperitoneal injection with a mix of ketamine (100 mg/kg) and atropine (1.5 mg/kg). Animals were sacrificed when necessary by intraperitoneal injection of pentobarbital (330 mg/kg). Experiment was performed three times, SP-D^-/-^ and SP-D^+/+^ infected-mice groups were composed of 13-16; 26-30; 32-26 animals, respectively. Control groups, SP-D^-/-^ and SP-D^+/+^ non-infected mice, were composed of 5 animals. Mice were housed in filtered boxes and maintained under specific pathogen-free conditions. Experiments were performed in accordance with national guidelines governing use of laboratory animals and were approved by the local animal care and use committee (Permit number 2007/33.1).

### 3.18. VACV-WR intranasal infection model

The infectious dose that was lethal to 50% of mice (LD_50_) was determined by infecting black Swiss SP-D^+/+^ mice (from NIH) with VACV-WR. Mice (five weeks old, 6 mice per group) were infected intranasally with 25 µL of the VACV-WR solution containing multiple dilutions of VACV-WR (1 x 10^5^, 5 x 10^5^ and 1 x 10^6^ PFU/mice). The LD_50_ of VACV-WR was calculated as 5 x 10^5 ^PFU. Purified virus was diluted in F12K medium (Gibco). Mice (five weeks old) were infected intranasally with 25 µL containing 1 LD_50_ and control groups were instilled with 25 µL of F12K medium. Groups of mice were monitored for mortality daily until 17 days post-infection (p.i).

### 3.19. Statistical analysis

For *in vitro* studies, the two-tailed unpaired Student’s t-test was performed. The animal survival study was analyzed with the Log-Rank test. Values of p<0.05 were considered significant.

## 4. Conclusions

We demonstrate in this study that SP-D plays an important role in pulmonary host defense against the variola virus. Using *in vitro* interactions, we show that SP-D blocked entry of VACV and could provide protection against infection *in vivo*. Binding to SP-D was mediated by viral protein A27; however the exact mechanism was not entirely elucidated and additional experiments would be undertaken to precisely determine the involvement of NCRD in the interaction. *In vivo* experiments also supported that SP-D was required for VACV infection. Further studies are needed to address the potential antiviral activity of SP-D and its capacity to modulate inflammatory responses.

## Abbreviations

A27: vaccinia virus A27 membrane protein (14-kDa fusion protein)
CRD: carbohydrate recognition domain
HA: hemagglutinin protein of IAV
IAV: *Influenza* A virus
MBL: mannan-binding lectin
NCRD: recombinant trimeric neck+CRDs
SP-A: surfactant protein A
SP-D: surfactant protein D
RSV: respiratory syncycial virus
VACV: vaccinia virus
VARV: variola virus
